# Associations Between the Nurse Work Environment and Job Outcomes Among Inpatient Psychiatric Mental Health Registered Nurses: A Cross‐Sectional Observational Study

**DOI:** 10.1111/jpm.70189

**Published:** 2026-07-31

**Authors:** Justinna Dixon, Hyunmin Yu, Ann Kutney‐Lee, J. Margo Brooks Carthon

**Affiliations:** ^1^ Center for Health Outcomes and Policy Research University of Pennsylvania School of Nursing Philadelphia Pennsylvania USA; ^2^ National Clinician Scholars Program University of Pennsylvania Perelman School of Medicine Philadelphia Pennsylvania USA; ^3^ Leonard Davis Institute of Health Economics University of Pennsylvania Philadelphia Pennsylvania USA; ^4^ Center for Healthcare Evaluation, Research and Promotion Corporal Michael J. Crescenz VA Medical Center Philadelphia Pennsylvania USA

## Abstract

**Introduction:**

Psychiatric Mental Health Registered Nurses (PMH‐RNs) are the largest group of healthcare providers working within inpatient mental health settings. Despite longstanding concerns about the emotional demands and safety of nurses practicing in these settings, few studies have examined associations between organizational factors and nurse job outcomes among inpatient PMH‐RNS.

**Aim/Question:**

To examine burnout, job dissatisfaction and intent to leave among PMH‐RNs employed in inpatient psychiatric units and how these job outcomes are associated with the quality of their nurse work environments.

**Method:**

This cross‐sectional study combined data from the 2023/2024 Penn Nurses4All and 2023 American Hospital Association (AHA) survey data.

**Results:**

Approximately 4 out of 10 inpatient PMH‐RNs experience high burnout as well as high levels of job dissatisfaction and intent to leave their roles. More favourable nurse work environments were associated with reduced negative job outcomes among inpatient PMH‐RNs.

**Discussion:**

Our findings provide insight into the current state of the PMH‐RN workforce and how the nurse work environment may represent a modifiable factor to improve nurse wellbeing, job satisfaction and job retention among this group. Policymakers and administrators should prioritize this vital group of health care professionals by improving interdisciplinary teamwork, promoting adequate staffing levels and creating a safe and inclusive environment for PMH‐RNs.

## Accessible Summary

**Originality (What the Paper Adds to Existing Knowledge):**

– Previous research has demonstrated that aspects of the environment in which nurses work play an important role in how nurses view their work and workplace and experience, yet no recent studies explore this in psychiatric nurses specifically.
– This paper shows that the nurse work environment plays an important role in how psychiatric nurses feel at work, such as whether they are burnt out, dissatisfied in their roles and planning to leave their roles.

**Significance (What Are the Implications or Impacts of the Paper):**

– Healthcare administrators and policymakers should prioritize improving organizational support for psychiatric nurses, such as promoting supportive administration and supervisors, having adequate levels of staff and fostering strong interdisciplinary relationships.

**Rigour (What Approach Was Taken to Ensure the Method Used Was Good Quality):**

– This study used several validated questionnaires to assess nurse job outcomes and the nurse work environment.
– Our study utilized data from a large, geographically diverse sample of U.S. psychiatric mental health nurses and combined data from multiple data sources.

**Relevance Statement:**

This study is directly relevant to the practice of mental health nursing as it relates to the current state of the psychiatric nursing workforce and factors related to the retention and well‐being of this population.

## 1 Introduction

Psychiatric mental health registered nurses (PMH‐RNs) play a central role in caring for patients with mental health conditions across inpatient settings (Hurley et al. 2022). Although the delivery of inpatient psychiatric care is highly interdisciplinary, PMH‐RNs comprise the largest portion of the inpatient psychiatric workforce in the U.S. and globally, comprising 44% of the mental health care workforce globally (Stewart et al. 2022). Specifically, there are over 120,000 practicing in the U.S. (US Bureau of Labor Statistics 2024). In these settings, inpatient PMH‐RNs are directly responsible for maintaining safe and therapeutic environments, administering medications, monitoring treatment response, leading group therapy sessions and managing acute substance withdrawal symptoms and behavioural crises (Hurley et al. 2022). In addition to the emotional demands of providing care to individuals in acute psychological distress, PMH‐RNs are increasingly delivering such care in the context of rising patient acuity (Mahgoub et al. 2023) and stagnant staffing levels (Liu et al. 2025; Staggs 2019). These combined effects may place PMH‐RNs at heightened risk for experiencing adverse job outcomes, such as burnout, job turnover and job dissatisfaction.

Burnout is a syndrome characterized by high emotional exhaustion, depersonalization and a diminished sense of personal accomplishment from work (Maslach and Leiter 2016). National organizations have identified burnout as a significant threat to clinician wellbeing, workforce sustainability and subsequently, the quality and safety of healthcare (National Academies of Sciences et al. 2019). High levels of burnout are particularly concerning for the nursing workforce, with over 30% of U.S. nurses experiencing significant burnout symptoms (Shah et al. 2021). An international meta‐analysis estimated that similar levels may be experienced among PMH‐RNs (López‐López et al. 2019). Burnout has been linked to numerous adverse job outcomes for nurses, including lower nurse retention and moral distress (Karakachian and Colbert 2019; Lasater 2024). Given the current shortage of PMH‐RNs, concerns have also been raised about nurses leaving their positions and being dissatisfied with their current roles (Stewart et al. 2022). Studies have qualitatively explored these outcomes (Ratnapradipa et al. 2024), however, there is limited quantitative evidence examining predictors of poor job outcomes among PMH‐RNs.

Prior research suggests that both individual and organizational factors contribute to burnout and related job outcomes among nurses generally. Individual characteristics such as age, education, and years of experience have been associated with burnout risk (Dall'Ora et al. 2020). Similarly, studies have found organizational factors within hospitals, such as workload and support from leadership, are also related to burnout and job outcomes (Meredith et al. 2022; Shah et al. 2021). Among these, the nurse work environment has emerged as a particularly influential factor. The nurse work environment is a multidimensional concept defined by organizational factors that either support or diminish a nurse's ability to deliver high‐quality care (Lake 2002). Multiple studies have documented clear associations between the nurse work environment and adverse nurse job outcomes; however, the bulk of existing studies have been conducted in general acute care settings (Aiken et al. 2023).

Despite longstanding concerns about the emotional demands and safety of nurses practicing in inpatient psychiatric settings (Jang et al. 2022), few studies have examined associations between the nurse work environment and adverse nurse job outcomes specifically among PMH‐RNs in the United States. To our knowledge, only one study conducted by Hanrahan et al. (2010) found that PMH‐RNs working in hospitals with higher quality work environments had lower levels of emotional exhaustion and depersonalization (Hanrahan et al. 2010). However, the findings of this study, conducted over 15 years ago in one state, likely do not reflect current practice conditions.

Therefore, our study aims to fill an important and timely gap in the literature. Here, we examine burnout, intent to leave and job dissatisfaction among PMH‐RNs employed in inpatient psychiatric units across 10 U.S. states. We also assess how these job outcomes are associated with the quality of their nurse work environments.

## 2 Methods

### 2.1 Study Design and Data Sources

This cross‐sectional study combined data from the 2023/2024 Penn Nurses4All and the 2023 American Hospital Association (AHA) Survey. The Penn Nurses4All Study provided data on nurse job outcomes and work environments across 10 U.S. states (CA, FL, IL, LA, NJ, NM, NY, OR, PA and WA) (Muir et al. 2025; Yu et al. 2026). Between December 2023 and March 2024, all actively licensed registered nurses from nine states were invited via email to complete the survey. There was no specific sampling method used to recruit nurses from specific hospitals or specialties; the survey targeted all nurses, yielding responses from nurses in various health care sectors (e.g., hospitals, long‐term care, school settings, primary care) as well as retired or unemployed nurses who maintained their nursing licence. In Pennsylvania, where only mailing addresses are available, 30% of registered nurses were randomly sampled and recruited through mail and electronic survey methods. A modified Dillman method was used to encourage study participation with follow‐up reminders that were sent at scheduled intervals to non‐respondents (Dillman et al. 2014). We utilized a criterion‐standard double‐sampling method with intensive follow‐up of nonresponders, which has demonstrated minimal evidence of nonresponse bias in previous iterations of this survey strategy (Lasater et al. 2019). To facilitate construction of hospital‐level estimates, respondents were provided a list of non‐federal, acute care hospitals and asked to indicate their current employer; those who worked at hospitals not on the list were asked to write in their hospital's name. Data on hospital characteristics were obtained from the 2023 AHA Annual Survey, a nationwide survey targeting all hospitals in the U.S. with a response rate averaging 70% across several survey iterations (American Hospital Association 2023).

### 2.2 Ethics Approval

All study procedures were approved by the Institutional Review Board (IRB protocol number: 850227). Nurses received information about the purpose of the survey, were informed that participation was voluntary and responses were anonymous and were advised that completion of the survey indicated informed consent.

### 2.3 Study Sample

To examine the association between hospital‐ level nurse work environment and job outcomes among inpatient PMH‐RNs, our study established the following inclusion criteria: (1) registered nurses working in hospitals and provided hospital names, (2) worked in hospitals with at least five nurse respondents, allowing us to generate reliable estimates of the nurse work environment and (3) nurses who had complete data on job outcomes. Based on the 2024 National Directory of Mental Health Treatment Facilities, our survey reached approximately 75% of inpatient psychiatric units across the 10 U.S states (Substance Abuse and Mental Health Services Administration 2024). Among the 113,811 nurses who completed the Penn Nurses4All survey, our final analytical sample includes 740 nurses in 297 general acute care or freestanding psychiatric hospitals. There were an average of 23 nurse respondents and an average of 3 PMH‐RN respondents per hospital. Figure 1 displays a flow diagram of sample selection from the 2024 Nurses4All survey showing the identification of nurses working in psychiatric units across 297 hospitals in 10 U.S. states.

**FIGURE 1:**
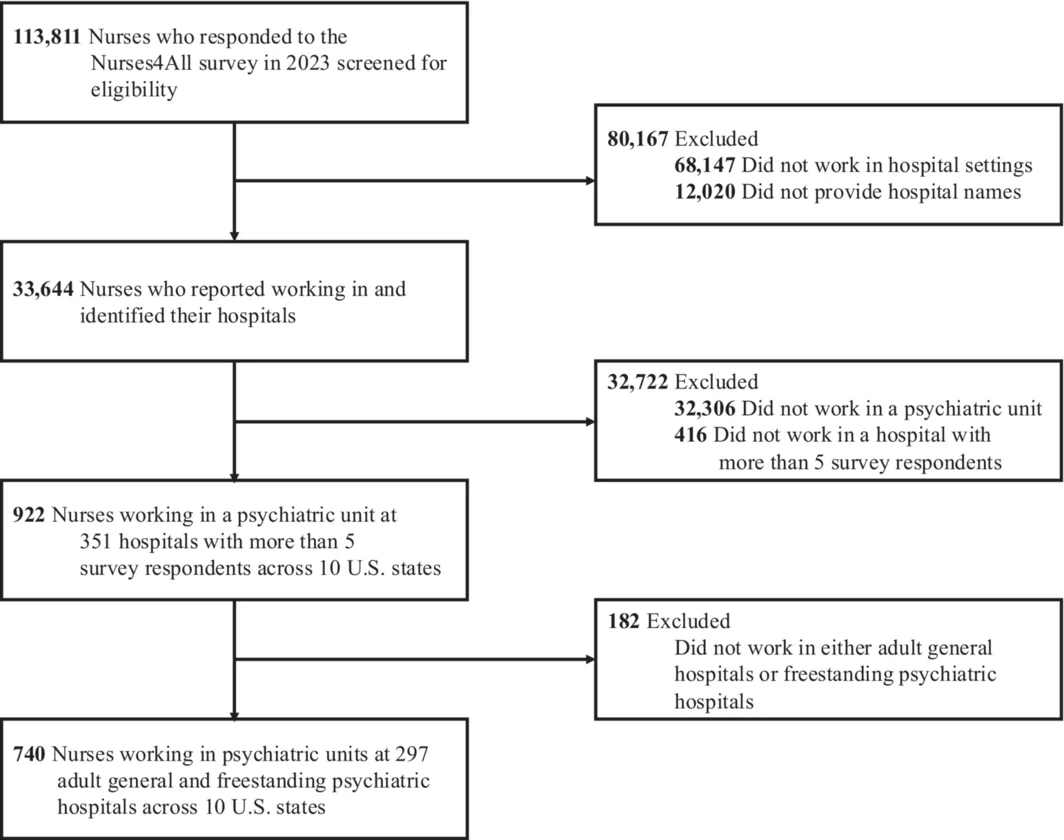
Sampling Diagram.

### 2.4 Study Variables

#### 2.4.1 Independent Variable: Nurse Work Environment

Our independent variable was the nurse work environment, measured by the PES‐5, the validated short form version of the Practice Environment Scale of the Nursing Work Index (PES‐NWI) (Lake et al. 2024). The PES‐5 uses one item from each of the five subscales from the original 31‐item PES‐NWI (nurses' participation in organizational affairs, nursing foundations for care quality, nurse manager leadership and support, nurse–physician relations, and staffing and resource adequacy) (Lake 2002, 2007). Each item's score reflects the extent to which a nurse agrees with statements that supportive traits are present and ranges from 1 (strongly disagree) to 4 (strongly agree), with higher scores indicating a more favourable work environment. The original developers of the PES‐5 evaluated several aspects of validity in the development of the instrument using a sample of registered nurses in the U.S. (Lake et al. 2024). Content validity of the PES‐5 was supported by expert panel evaluations, which ranked the selected items highly within their respective subscales and correlated with the original PES‐NWI. Construct validity was established by correlating the score of the short form PES‐5 to the original PES‐NWI and conducting a confirmatory factor analysis of the five‐item PES‐5 (Lake et al. 2024). Criterion validity was established through analyses of variance comparing mean PES‐5 scores across responses to a single‐item measure of the work environment.

The original developers of the PES‐5 also evaluated two types of reliability. Cronbach's alpha, which is a measure of internal consistency, and the intraclass correlation, ICC (1, *k*), which demonstrates the stability of the PES‐5 score for clustered data (Lake et al. 2024). In their evaluation, the Cronbach's alpha was 0.81, while the ICC (1, *k*) was 0.89.

The Cronbach's alpha of the PES‐5 in our sample was 0.8. We computed individual nurse‐level mean scores on the PES‐5 for those who completed all scale items, then aggregated them at the hospital level by averaging the scores of all nurses within each hospital. We used the hospital‐level mean composite score for the PES‐5 for the main analyses, and hospital‐level mean scores for each individual scale item for sensitivity analyses exploring which work environment domains drive relationships between the work environment and job outcomes. The intraclass correlation coefficient, ICC (1, *k*), which reflects the degree of response agreement among respondents within a hospital compared to the agreement between hospitals, was 0.70 in our sample. A value above 0.60 is considered satisfactory for cluster aggregation (Glick 1985).

#### 2.4.2 Dependent Variables: PMH‐RN Job Outcomes

Our dependent variables were three job outcomes among PMH‐RNs: burnout, intent to leave and job dissatisfaction. Burnout was assessed using the Emotional Exhaustion Scale of the Maslach Burnout Inventory (Maslach et al. 1997). This instrument has been highly used and validated in a variety of samples of healthcare professionals in the U.S., especially registered nurses (Beckstead 2002; Dyrbye et al. 2017). Several studies have established various aspects of the validity of the MBI, including its predictive and convergent validity (De Beer et al. 2024; Maslach and Jackson 1981). Nurses were classified as experiencing ‘high burnout’ if their score on the subscale was 27 or higher, corresponding with the instrument's guidelines (Maslach et al. 1997). The Cronbach's alpha in this sample was 0.95, indicating strong internal consistency.

Intent to leave was derived from a single‐item question, ‘Do you plan to be with your current employer one year from now?’ This variable was coded as 0 for ‘yes’ and 1 for ‘no’. Job dissatisfaction was measured using a single‐item question, ‘How satisfied are you with your primary job?’ Responses were recorded using a four‐point Likert scale, with ‘very dissatisfied’ or ‘a little dissatisfied’ being classified as ‘dissatisfied’, consistent with previous studies (Iroegbu et al. 2025; Muir et al. 2024).

### 2.5 Covariates

We accounted for both nurse‐level and hospital‐level characteristics based on empirical and theoretical relevance.

#### 2.5.1 Nurse‐Level Covariates

Nurse‐level covariates included age, gender, years of experience in the current hospital, race and ethnicity and level of education. Nurses' age and years of experience in their current hospital were treated as continuous variables. Gender was a binary variable with responses ‘male’ and ‘female’. Race and ethnicity were classified into five categories: ‘Non‐Hispanic Asian’, ‘Non‐Hispanic Black’, ‘Non‐Hispanic White’, ‘Hispanic’ and ‘Multi‐racial or other’. Nurses' level of education was dichotomized into two options: ‘associate's degree or lower’ and ‘bachelor's degree or higher’. These were selected to be included in our model based on their documented association with job outcomes (Dall'Ora et al. 2020; Gómez‐Urquiza et al. 2017).

#### 2.5.2 Hospital‐Level Covariates

Hospital‐level covariates were obtained from the AHA Annual Survey. There are significant differences in the physical environment and personnel availability and training across these settings, which may influence how nurses experience working in these settings and their propensity to experience poor job outcomes (Johnson et al. 2022). Hospital covariates included hospital type (i.e., general acute, freestanding psychiatric), teaching status, hospital size and ownership status. Teaching status reflects the potential variation in staffing models, documentation standards and other institutional policies across teaching and non‐teaching hospitals (Meredith et al. 2022). Teaching status was determined by calculating the ratio of resident physicians and fellows to the number of hospital beds. Hospitals were categorized into three groups: nonteaching, minor teaching (a ratio of 1:4 or less) and major teaching (a ratio greater than 1:4) (Ayanian et al. 1998). Hospitals were categorized by size into three groups based on previous literature: small (100 beds or fewer), medium (101 to 250 beds) and large (over 250 beds) (Aiken et al. 2002). Ownership status was classified as federal, not‐for‐profit and for‐profit.

### 2.6 Statistical Analysis

Statistical analyses were conducted in R (version 4.5.2). We conducted descriptive analyses to describe the sample of PMH‐RNs working in inpatient psychiatric units, their job outcomes, and the characteristics of the hospitals they worked at. We reported on the means and standard deviations (SDs) for continuous variables and frequencies and percentages for categorical variables.

Because nurses were nested within hospitals, we used multilevel logistic regression to account for clustering and shared contextual influences within hospitals, which could otherwise violate the independence assumption and bias standard errors. To examine associations between nurse‐level variables (age, years of experience in the current hospital, gender, education and race and ethnicity) and hospital‐level variables (nurse work environment, hospital type, teaching status, size, ownership) with nurse job outcomes, we specified a two‐level model with a hospital random intercept. We first estimated a null model with only the outcome variable, followed by a model including nurse‐level variables and then a full model incorporating both nurse‐ and hospital‐level variables. For the main analysis, the work environment was included using hospital‐level mean scores of the composite PES‐5. In sensitivity analyses, hospital‐level mean scores for each individual PES‐5 item were used.

Regarding missing data, a significant proportion of respondents had missing data for nurse‐level covariates. Although the exact mechanisms underlying the missingness could not be definitively determined, exploratory analyses suggested that the missingness was related to observed variables. Therefore, we assumed that the data were missing at random (MAR) and applied multiple imputation using the *mice* R package (van Buuren and Groothuis‐Oudshoorn 2011; White et al. 2011).

## 3 Results

### 3.1 Sample Characteristics

Table 1 provides information on the characteristics of our analytic sample of 740 inpatient PMH‐RNs. The mean age of our sample was 50.3 years (SD = 12.7), and the average years of experience within their current hospital was 8.9 years (SD = 9.0). Most respondents were female (79.7%) and held a bachelor's degree or higher (66.9%). The majority of the sample identified as non‐Hispanic White (64.3%). In unadjusted analyses, approximately 38% of inpatient PMH‐RNs in our sample reported experiencing high burnout. Additionally, 22.0% reported intending to leave their job within the next year, and 29.5% reported being dissatisfied with their current role.

**TABLE 1:** Characteristics of the Inpatient Psychiatric Registered Nurse Sample (*N* = 740).

Age (years), Mean (SD)	50.3 (12.7)
Years of Experience in Current Hospital, Mean (SD)	8.9 (9.0)
Female, *n* (%)	590 (79.7)
Bachelor's degree or higher, *n* (%)	495 (66.9)
Race and Ethnicity, *n* (%)	
Non‐Hispanic Asian	75 (10.1)
Non‐Hispanic Black or African American	87 (11.8)
Non‐Hispanic White	476 (64.3)
Hispanic	55 (7.4)
Multi‐racial or Other	47 (6.4)
Job Outcomes	
High Burnout, *n* (%)	282 (38.1)
Intent to Leave, *n* (%)	163 (22.0)
Job Dissatisfaction, *n* (%)	218 (29.5)

Abbreviation: SD, standard deviation.

Table 2 describes the characteristics of the 297 hospitals included in our sample. The majority of our sample was general acute care hospitals (90.2%) as compared to freestanding psychiatric hospitals (9.8%). Almost half were classified as minor teaching hospitals (49.2%). The majority of hospitals were not‐for‐profit (80.1%) and large in size with more than 250 beds (58.6%). The mean hospital‐level composite PES‐5 score was 2.64 (SD = 0.28). Among the PES subscales, the highest mean score was for nurse–physician relationships (3.16, SD = 0.23), whereas the lowest was for staffing adequacy (2.23, SD = 0.42).

**TABLE 2:** Characteristics of hospital sample (*N* = 297).

	*n* (%)
Hospital Type	
General Acute	268 (90.2)
Free‐Standing Psychiatric	29 (9.8)
Teaching Status	
Major	78 (26.3)
Minor	146 (49.1)
None	73 (24.6)
Hospital Size	
Small (< 100 beds)	25 (8.4)
Medium (100–250 beds)	98 (33.0)
Large (> 250 beds)	174 (58.6)
Ownership	
For‐Profit	28 (9.4)
Not‐for‐Profit	238 (80.2)
Federal	31 (10.4)
Nurse Work Environment (PES‐5)	Mean (SD)
Composite PES‐5 score^a^	2.64 (0.28)
Administration that listens and responds to nurse concerns^a^	2.32 (0.37)
A supervisor who is a good manager and leader^a^	2.81 (0.32)
Physicians and nurses have good working relationships^a^	3.16 (0.23)
Enough staff to get work done^a^	2.23 (0.42)
A clear philosophy of nursing that pervades the patient care environment^a^	2.68 (0.37)

Abbreviations: PES, practice environment scale; SD, standard deviation.

^a^ This score ranges from 1 to 4.

### 3.2 Association of Work Environment With Burnout, Intent to Leave and Job Dissatisfaction

Table 3 displays the results of multilevel logistic regression models examining the association between nurse work environment and job outcomes among inpatient PMH‐RNs. After adjusting for both nurse and hospital‐level characteristics, we found that for every 1‐point increase in the nurse work environment, there is an approximately 70% decrease in the odds of experiencing high burnout (adjusted odds ratio [aOR] = 0.30, 95% CI = 0.15–0.58). Similarly, a 1‐point increase in the nurse work environment is associated with a 53% decrease in the odds of intending to leave their jobs within a year (aOR = 0.47, 95% CI = 0.23–0.98). Additionally, a 1‐point increase in the nurse work environment is associated with 59% decrease in the odds of experiencing job dissatisfaction (aOR = 0.41, 95% CI = 0.20–0.82).

**TABLE 3:** Odds ratios demonstrating the associations between work environment and PMH‐RN job outcomes (*n* = 740 nurses).

	Burnout		Intent to leave		Job dissatisfaction	
	aOR (95% CI)	*p*	aOR (95% CI)^a^	*p*	aOR (95% CI)	*p*
Composite PES‐5 score^a^	0.30 (0.15–0.58)	< 0.001	0.47 (0.23–0.98)	0.04	0.41 (0.20–0.82)	0.01
Administration that listens and responds to nurse concerns^a^	0.48 (0.29–0.79)	0.01	0.59 (0.34–1.04)	0.07	0.58 (0.35–0.96)	0.03
A supervisor who is a good manager and leader^a^	0.55 (0.32–0.94)	0.03	0.55 (0.30–1.00)	0.05	0.61 (0.35–1.04)	0.07
Physicians and nurses have good working relationships^a^	0.40 (0.18–0.88)	0.02	0.87 (0.35–2.13)	0.76	0.72 (0.32–1.66)	0.45
Enough staff to get work done	0.44 (0.29–0.68)	< 0.001	0.60 (0.37–0.96)	0.03	0.54 (0.38–0.85)	0.01
A clear philosophy of nursing that pervades the patient care environment	0.49 (0.31–0.81)	0.01	0.65 (0.37–1.14)	0.13	0.53 (0.31–0.90)	0.02

Abbreviations: aOR, adjusted odds ratios; CI, confidence interval; PMH‐RN, Psychiatric Mental Health Registered Nurse.

^a^ Models controlled for nurse characteristics (age, gender, education level, years of experience in current hospital and race and ethnicity) and hospital characteristics (type, size, teaching status and ownership type).

In sensitivity analyses examining the associations of each PES‐5 domain separately rather than the composite PES‐5 score, we found that higher scores in all five domains were significantly associated with burnout. However, staffing adequacy, defined as nurses having enough staff to get the job done, was the only domain significantly associated with all three job outcomes.

## 4 Discussion

Our study found that approximately 4 out of 10 inpatient PMH‐RNs experience high burnout. This is comparable to national averages of burnout among RNs in the United States. (Aiken et al. 2023; Shah et al. 2021), but notably higher than the recent international estimate of 25% among PMH‐RNs (López‐López et al. 2019). Additionally, we found that 22% of inpatient PMH‐RNs intend to leave their jobs within 1 year, and almost one in three nurses reported being dissatisfied with their jobs. Our study results also suggest that the nurse work environment is significantly associated with job outcomes for PMH‐RNs.

There was notable variation in these outcomes by the quality of the nurse work environment, with a significant inverse association between the nurse work environment and job outcomes among PMH‐RNs. Specifically, for every one‐point increase in the work environment, akin to a one‐unit increase in agreement, such as from agree to strongly agree, there were significant decreases in the odds of PMH‐RNs experiencing burnout, intending to leave their jobs, or being dissatisfied with their current roles. Our sensitivity analysis revealed that staffing adequacy was a strong driver of this relationship for all three job outcomes. The results of our study also indicate that other domains of the nurse work environment, such as the interdisciplinary relationships between physicians and nurses, leadership skills of their supervisors and responsiveness of administration, were related to experiences of burnout, job dissatisfaction and poor retention among PMH‐RNs.

Our findings provide insight into the current state of the PMH‐RN workforce and how the nurse work environment may represent a modifiable factor to improve nurse wellbeing, job satisfaction and job retention among this group. Retention of the current PMH‐RN workforce is particularly vital as the American Psychiatric Nurses Association (APNA) estimates that almost half of the PMH‐RN workforce plans to retire within the next 10 years (American Psychiatric Nurses Association 2022). This is cause for concern as there is already a significant mental health provider shortage, with less than one‐third of U.S. counties (26.4%) having an adequate supply of mental health professionals (KFF 2024). We also found that the staffing adequacy was the lowest‐rated aspect of the nurse work environment. This finding is highly aligned with recent evaluations noting that staffing has not adequately kept pace with the growing demand for mental health care (Staggs 2019).

### 4.1 Implications for Policy, Practice and Research

Our findings have several implications for mental health care policy, practice and research. PMH‐RNs are uniquely vulnerable to experiencing high emotional burden due to the intense emotional nature of interactions with patients on inpatient units. Compared to other specialties, inpatient PMH‐RNs are more likely to manage physically threatening, difficult, or demanding patients (Itzhaki et al. 2015). Workplace violence and safety concerns are major challenges for PMH‐RNs that can contribute to high burnout and low retention (Ratnapradipa et al. 2024). Unsupportive work environments in which there are significant organizational issues, such as incivility among colleagues and poor perceptions of leadership competence, can further contribute to psychological distress among PMH‐RNs (Wei et al. 2018).

Promoting a healthy work environment for PMH‐RNs is especially crucial as literature has demonstrated that, on average, PMH‐RNs have higher levels of engagement and a sense of buy‐in with their organizations than other specialties (Curran et al. 2020). This strong engagement among PMH‐RNs reflects their dedication to their roles but could be diminished in the face of unsupportive nurse managers. Our findings suggest that the work environment leadership domain had a pronounced relationship with burnout and job dissatisfaction, which signals the value of support provided by leadership. Previous work has put forth the need for effective nursing leadership, communication, teamwork and professional autonomy as major pillars to develop healthy nurse work environments (Mabona et al. 2022). Specifically, several nursing practice guidelines highlight the significant duty of nursing leadership to ensure a physically and psychologically safe environment to promote healthy work environments (Mabona et al. 2022). Additionally, a recent systematic review of 17 articles found that nurses with greater supervisor support and leaders with empowering styles of leadership had overall better work‐related well‐being, including reduced burnout (Niinihuhta and Häggman‐Laitila 2022). Our findings similarly suggest that healthcare leadership is a critical component to fostering a safe and responsive culture for all nurses and should be leveraged for the well‐being of PMH‐RNs.

Our findings also suggest that improved staffing levels represent an important opportunity to support the well‐being of PMH‐RNs. Insufficient staffing levels may diminish the ability of PMH‐RNs to provide care to the fullest extent of their training and heighten safety threats in the inpatient psychiatric setting. Additionally, changes in patient demand and acuity warrant new staffing models that align with both PMH‐RN and patient needs (Johnson et al. 2023).

Lastly, future research should aim to describe the barriers and facilitators of PMH‐RN wellbeing and retention from both PMH‐RN and administrative perspectives. Healthcare administrators can add a unique understanding of structural aspects of care delivery that influence PMH‐RN outcomes. Researchers should also use qualitative methods to explore the reasons why PMH‐RNs are experiencing high levels of burnout, dissatisfaction and turnover to gain a clear understanding of individual and organizational influences on this group of healthcare professionals.

### 4.2 Limitations

Our study has limitations. First, due to the cross‐sectional design, we are unable to determine a causal relationship between work environment and job outcomes. Additionally, although our sample was from 10 geographically diverse U.S. states, findings may not be generalizable to PMH‐RNs in other states. Although the PES‐5 is a validated composite measure of the work environment, its five items have not been independently validated as indicators of distinct domains of the work environment.

## 5 Conclusion

Negative job outcomes such as high burnout, intent to leave and job dissatisfaction are common among PMH‐RNs. Optimizing work environments is a potential leverage point to improve satisfaction and retention among the largest group of inpatient mental healthcare professionals. Policymakers and administrators should prioritize this vital group of healthcare professionals by improving interdisciplinary teamwork, promoting adequate staffing levels and creating a safe and inclusive environment for PMH‐RNs.

## Funding

J.D.'s effort was supported by the National Institutes of Health, National Institute for Nursing Research T32 training (T32NR007104), Advanced Training in Nursing Outcomes Research and the National Clinician Scholars Program. The data in this study were produced with support from the National Institute of Nursing Research (Project Number R01NR014855 PI: McHugh).

## Conflicts of Interest

The authors declare no conflicts of interest.

## Data Availability

The data that support the findings of this study are available on request from the corresponding author. The data are not publicly available due to privacy or ethical restrictions.
